# Bio-Activated PEEK: Promising Platforms for Improving Osteogenesis through Modulating Macrophage Polarization

**DOI:** 10.3390/bioengineering9120747

**Published:** 2022-12-01

**Authors:** Haobu Chai, Wenzhi Wang, Xiangwei Yuan, Chen Zhu

**Affiliations:** 1 Department of Orthopaedics, The First Affiliated Hospital of University of Science and Technology of China, University of Science and Technology of China, Hefei 230001, China; 2 Department of Orthopaedics, Shanghai Jiao Tong University Affiliated Sixth People’s Hospital, Shanghai Jiao Tong University, Shanghai 200233, China

**Keywords:** polyetheretherketone, biomaterial modification, osteogenesis, macrophage polarization, osteoimmunomodulation

## Abstract

The attention on orthopedic biomaterials has shifted from their direct osteogenic properties to their osteoimmunomodulation, especially the modulation of macrophage polarization. Presently, advanced technologies endow polyetheretherketone (PEEK) with good osteoimmunomodulation by modifying PEEK surface characteristics or incorporating bioactive substances with regulating macrophage polarization. Recent studies have demonstrated that the fabrication of a hydrophilic surface and the incorporation of bioactive substances into PEEK (e.g., zinc, calcium, and phosphate) are good strategies to promote osteogenesis by enhancing the polarization of M2 macrophages. Furthermore, the modification by other osteoimmunomodulatory composites (e.g., lncRNA-MM2P, IL-4, IL-10, and chitosan) and their controlled and desired release may make PEEK an optimal bio-activated implant for regulating and balancing the osteogenic system and immune system. The purpose of this review is to comprehensively evaluate the potential of bio-activated PEEK in polarizing macrophages into M2 phenotype to improve osteogenesis. For this objective, we retrieved and discussed different kinds of bio-activated PEEK regarding improving osteogenesis through modulating macrophage polarization. Meanwhile, the relevant challenges and outlook were presented. We hope that this review can shed light on the development of bio-activated PEEK with more favorable osteoimmunomodulation.

## 1. Introduction

In clinic, bone losses and defects are always caused by traumatic fracture, osteolysis, bone-involved tumor resection, aseptic or infected osteonecrosis, spinal fusion, periodontitis, etc., and are typically repaired and improved by using the advanced osteogenic biomaterials by surgeries. In these processes, effective bone regeneration is important for prognosis [1,2,3,4,5,6]. In addition, the development of these biomaterials, for decades, has focused on the role that biomaterials play in directly regulating the osteogenic differentiation of the osteoblastic lineage cells, such as osteoblasts, osteoclasts, and bone mesenchymal stem cells (BMSCs) [7,8,9]. Therefore, a series of advanced osteogenic biomaterials have been developed to gain excellent osteogenesis by carrying out various strategies, such as the desired fabrication of biomaterials themselves (from two-dimensional to three-dimensional structure, from hydrophobic to hydrophilic surface, from nano to micro even macro size, etc.) [10,11,12], and the advanced integration between biomaterials and bioactive substances (from metallic to non-metallic elements, from inorganic to organic compounds, from genes to proteins, etc.) [13,14,15,16,17]. Based on this fruitful theory research, the application of these biomaterials seems to have a promising medical prospect.

However, it is very disappointing that the majority of these biomaterials are not used in clinic. Of the possible reasons, one is that we only focus on the direct relationships between the properties of biomaterials and the osteogenic differentiation of the osteoblastic lineage cells but neglect the role of other cells from other systems (such as the immune cells) during the process of bone formation and healing [18,19,20,21,22]. It is concluded that a study is probably imperfect or outdated if only following the traditional principles of enhancing osteogenesis. Therefore, an advanced osteogenic biomaterial could be better fabricated and put into clinical use when the bone biologies of all system cells are taken into consideration as much as possible.

As we know, when biomaterials are implanted into host, the immune system instantly recognizes the foreign bodies, the immune cells are activated, and a series of immune responses are triggered in turn [23,24,25]. An undesirable immune response could lead to the failure of the implantation by negatively influencing the bio-behavior of bone cells to compromise osseointegration between the implant and host bone [26,27]. In other words, the immune cells play an important role in the development of host bone, and there is a close crosstalk between the skeletal system and the immune system, which is linked by a series of cytokines, signaling molecules and proteins, receptors, etc. [28,29,30,31]. Of the immune cells, the biological behavior of macrophages is regarded as a critical factor because the different polarization phenotypes of macrophages result in different effects on the modulation of osteogenesis [28,29,32,33]. Therefore, in order to create a desirable environment of macrophage polarization for improving osteogenesis, some studies on developing biomaterial implants have been carried out [18,25,34].

As one of biomaterial implants, polyetheretherketone (PEEK) is a semi-crystalline, non-resorbable, high-performance, and thermoplastic material, and is being paid more and more attention in trauma, orthopedic, and spinal surgeries owing to its several excellent advantages [35,36,37,38,39,40]. For instance, there is a similar elastic modulus between PEEK (3–4 Gpa) and human cortical bone (18 Gpa), which contributes to reducing stress shielding. Because of its radiolucency, the new bone formation surrounding the PEEK implant can be better evaluated through imageological examinations (such as X-ray) during the postoperative follow-ups. Additionally, biochemical corrosion can be avoided when PEEK is implanted into the host because of its environmental resistance. However, the bio-inertness of pure PEEK, to some extent, limits its application in the field of medicine. In order to improve the bioactivity of PEEK, a porous surface, on one hand, usually needs to be developed. The porous structure not only benefits the induction of shifts in local macrophage polarization but is only used as a carrier where some bioactive substances are loaded [41]. Presently, in order to combine with the bioactive substances to improve its osteogenesis through polarizing macrophage, the porous structure on the surface of PEEK can be fabricated through several special techniques (see Table 1). On the other hand, bioactive substances can also be combined with pure PEEK through some special intermedi, such as catecholamines which include dopamine and norepinephrine (NE). Furthermore, in weakly alkaline solution, these intermedi polymerize to form small aggregates which can bind to a variety of materials including ceramics, metals, and polymers [42,43].

With the development and design of PEEK, it has been proved that PEEK is fabricated into the porous implant or used as a carrier loading some bioactive substances (e.g., zinc and calcium) to improve the osteogenic process by inducing a shift of local macrophage polarization in the foreign body reaction, and the use of them has extensively increased [18,41]. To better understand the relationships among PEEK, macrophage polarization, and osteogenesis, we retrieved the featured research on this issue and detailed several important topics. Firstly, the synthesis and characteristics of porous PEEK were addressed briefly through reviewing previous studies. Secondly, the relationship between host osteogenesis and macrophage polarization was detailed. Thirdly, the enhanced osteogenesis of bio-activated PEEK through inducing M2 macrophage polarization was emphasized. Finally, challenges and outlook were summarized.

## 2. Fabrication and Characteristics of Porous PEEK

**Figure 1:**
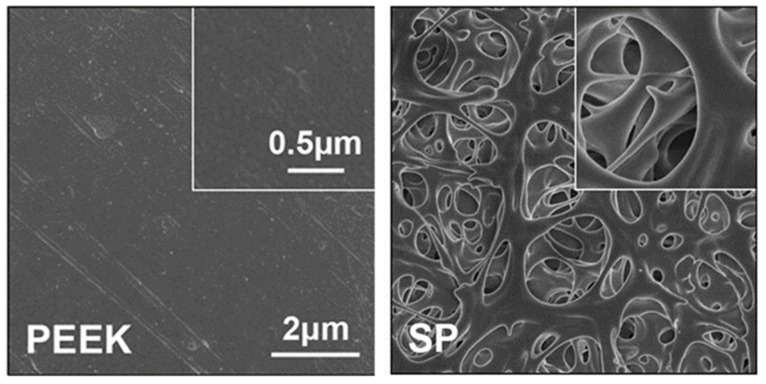
Surface morphology of PEEK and sulfonated PEEK (SP) detected by SEM. Reprinted with permission from Ref. [57]. Copyright 2022, Elsevier.

**Figure 2:**
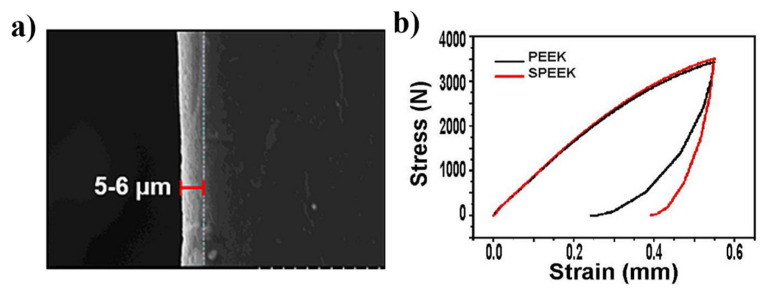
(**a**) Thickness of the sulfonation layer (5–6 μm). (**b**) Stress–strain curve for PEEK and SPEEK. Reprinted with permission from Ref. [57]. Copyright 2022, Elsevier.

Just as described in Table 1, a porous structure on PEEK surface can be fabricated through special physio-chemical techniques or computer techniques. Take the sulfonation technique for example: PEEK is first immersed in concentrated sulfuric acid (about 98%) while being stirred through magnetic stirring machine in order to fabricate a uniform porous surface. Next, the residues of sulfuric acid on PEEK surface are gotten rid of through immersion in deionized water for 10 min. Then, the sulfur concentration is decreased as much as possible through a hydrothermal treatment (120 °C for 4 h). Finally, the sulfonated PEEK (SPEEK) is gently washed using deionized water and dried at room temperature. According to the results of our work [57], Figure 1 shows the SEM images of PEEK before and after sulfonation. On the other hand, according to the results of our previous study (Figure 2), the thickness of the porous layer is about 5–6 μm after PEEK is sulfonated, meaning it is too thin to compromise its bulk mechanical properties, which is proved by the results of the mechanical tests that the porous surface is not destroyed even if pressed down by external force and the results of the stress–strain curve which show that their mechanical properties (e.g., breaking point, elasticity modulus, and fracture strength) are similar between PEEK and sulfonated PEEK (Table 2). This is consistent with the results from previous studies [49,50].

## 3. Development of Bio-Activated PEEK 

With regard to the techniques of combination between the bioactive substances and PEEK, they can be generally divided into three categories. (1) Direct surface modification: plasma surface treatment; wet chemical treatment; ultraviolet/ozone surface treatment; accelerated neutral atom beam surface treatment; nanofiber fabrication; laser surface modification [58,59,60,61,62,63,64,65]. (2) Deposition techniques: vacuum plasma spray; chemical deposition; plasma spray; arc ion plating; radio frequency magnetron sputtering; electron beam deposition [66,67,68,69,70,71]. (3) Fabricating bioactive composites: selective laser sintering; injection molding; in situ synthetic method [72,73,74]. Though the preparation methods of combination between the bioactive substances and PEEK are significantly different, all designs and preparations require a win–win combination that not only can PEEK keep its own excellent properties, but that it also plays a desirable and durable role in host osteogenesis through the controlled release of the bioactive substances.

## 4. Macrophage Polarization for Osteogenesis

Of all the immune cells, macrophages presently have received a large amountof attention because of their high plasticity and different functional phenotypes in the period of the interaction between implants and host. In response to the local micro-environment signals caused by implants, macrophages can be polarized into different macrophage phenotypes which are mainly divided into M1 (classically activated/inflammatory phenotype) and M2 (alternatively activated/regenerative phenotype), just as T helper cells are named as Th1 and Th2 [75]. Briefly, lipopolysaccharide (LPS), interferon-γ (IFN-γ), and tumor necrosis factor-α (TNF-α) can induce macrophages into M1 polarization with anti-tumoral and pro-inflammatory properties, whereas macrophage exposure to the immune complexes, interleukin-4 (IL-4) and IL-10, can transform into M2 polarization, which has an anti-inflammatory property and improves tissue repair [76]. On the other hand, when exposed to different stimulating signals, M2 macrophage can be divided into M2a (induced by IL-13 and IL-4), M2b (induced by toll-like receptors (TLRs) or IL-1R agonists and immune complexes), and M2c (induced by IL-10) which has different functions including Th2 inflammation, allergy, immunoregulation, tissue repair, etc. [76]. Because macrophages can be switched into different functional phenotypes when responding to the local environment signals, it may be feasible that a desirable osteogenesis could be enhanced by regulating and controlling macrophage phenotypes.

Presently, in view of the different functions of M1 and M2 macrophages, there is a general consensus that the M2 phenotype is more favorable to improve osteogenesis when compared to M1 phenotype. It is well known that M1 macrophage secretes a series of proinflammatory cytokines (IL-6, IL-1β, and TNF-α) and proteinase (matrix metalloproteinases (MMPs)) which could accelerate the activity of osteoclasts, and results in bone resorption and the subsequent aseptic loosening of implants [77]. Nevertheless, M2 macrophage can express and secrete some helpful factors which induce and promote osteogenesis, such as vascular endothelial growth factor (VEGF), transforming growth factor-β (TGF-β), and, especially, bone morphogenetic protein-2 (BMP-2) [28,34,78]. Therefore, it is suggested that M2 macrophage plays an indispensable role in improving osteogenesis, and this point paves a new way for the development of the innovative biomaterial implants which not only directly focus on osteoblastic lineage cells but also emphasiz the role of macrophage polarization in osteogenesis.

When implanted into host bone, the innovative biomaterial is recognized as a foreign body by the host immune system, arousing immune responses and activating immune cells (e.g., macrophages). Then, macrophages, in response to the biomaterial implants, could be regulated and induced into M2 phenotype. The latter further produces a series of osteoinductive and osteogenic factors (TGF-β, VEGF, BMP-2, etc.) to enhance osteogenesis around implants. This effect is attributed to the shared mechanisms and cross-talks between immunology and osteology, which is probably defined as osteoimmunomodulation [79]. Based on the understanding of both systems, a new perspective will probably shed light on for the design and development of medical biomaterials (e.g., PEEK) to significantly improve osteogenesis through regulating a required macrophage polarization.

## 5. Bio-Activated PEEK for Osteoimmunomodulation

Presently, there are a great many studies which have demonstrated that the immune environment for inducing osteogenesis generated by biomaterial implants can be modulated through their surface characteristics and bioactive composites, such as topography, porosity, wettability, released ions, etc. For example, Chen and coworkers reported that nano-topography of biomaterials could modulate the immune response to improve osteogenesis surrounding the implants through possible underlying mechanisms [80,81]. This is consistent with the results of the previous study [82]. On the other hand, Lee CH et al. provided similar insight into the surface modulation of biomaterials that divalent cation chemistry (e.g., calcium and strontium) could regulate the cell shapes of adherent macrophages and significantly up-regulate the expression of M2 macrophage phenotypes when combined with the nanostructured titanium implant surfaces [83]. However, studies on bio-activated PEEK with improving osteogenesis by modulating macrophage polarization are very few. It is necessary to make a summary by reviewing relevant references to guide the osteoimmunomodulatory design and the clinical application of PEEK.

### 5.1. Hydrophilicity-Modified PEEK

Of the biomaterial surface characteristics, wettability plays an important role in the modulation of host cell adhesion, proliferation, and differentiation, and many studies showed that, on the highly hydrophobic biomaterial surface, proteins are adsorbed in denatured and rigid forms which inhibit cell adhesion; whereas, on the highly hydrophilic biomaterial surface, the proteins are prevented from adsorption or bound very weakly which leads to a higher cell adhesion and modulates cell bio-behavior [82,84,85,86]. Therefore, it is probably feasible that surface modification endows PEEK with a hydrophilic surface which induces a higher M2 macrophage polarization to improve the osteogenic process. As suggested by Lv L [87], when compared with the hydrophobic surface, the hydrophilic surface could enhance the anti-inflammatory and pro-healing performances of macrophages, which is attributed to the mechanism by which, on the hydrophilic surface, integrin β1 interacts with the adsorbed fibronectin (FN) and then stimulates macrophages into M2 phenotype, likely through the PI3K/Akt signaling pathway, while integrin β2 interacts with fibrinogen (FG) absorbed on the hydrophobic surface and, next, induces a higher M1 macrophage phenotype, probably via activating the NF-κB signaling pathway. 

Through surface plasma treatment and phosphorylation, the PEEK surface is endowed with hydrophilicity which triggers M2 polarization [58]. This is mainly because the hydrophobic surface can induce protein denaturation, and the latter stimulates macrophages to produce pro-inflammatory cytokines and nitric oxide which are the typical markers of M1 macrophages [88,89,90]. On the other hand, it is suggested that a hydrophilic surface is more important for enhancing M2 polarization when compared to the roughness of the PEEK surface [91]. However, the roughness of the biomaterial surface contributes to improving osseointegration between host bone and the implant [92,93]. Therefore, for the sake of the optimized osseointegration, the biomaterial surface characteristics (e.g., roughness and wettability) need to be thoroughly taken into account.

### 5.2. Zinc-Modified PEEK

It is well known that zinc is classified as one of the micro-nutrients in the essential trace elements and also the only metal that is an important component in at least 3000 proteins and six classes of enzymes, including transferases, isomerases, hydrolases, oxidoreductases, lyases, and ligases, and plays a vital role in the maintenance of internal environment homeostasis, such as reproductive health, physical growth and development, neuro-behavioral development, sensory function, and functioning of immune system [94,95,96,97]. 

In terms of zinc ions for improving osteoimmunomodulation, it has been well documented that the immune regulation of zinc is probably regulated by the NF-κB signaling pathway. That is, zinc can induce the expression of A20 zinc finger protein and the latter plays a positive role in the anti-inflammatory response through inhibiting the NF-κB canonical signaling pathway in macrophages [98,99]. In addition, Ooi TC et al. suggested that this anti-inflammatory effect of zinc is also attributed to the activated Nrf2/HO-1 signaling pathway, but independent of the MAPKs signaling pathway [100,101]. Its anti-inflammatory effect could result from the increase in M2 polarization and the decrease in M1 polarization. For instance, Liu and his partners evaluated the immunomodulatory function for guiding cell fate and bone regeneration among PEEK, porous PEEK, and zinc-coated porous PEEK, and their results showed that, when compared with PEEK, zinc-coated porous PEEK, mainly owing to the controllable release of zinc, could significantly induce macrophages into an anti-inflammatory phenotype (M2 macrophage); the latter then produced a set of osteogenic cytokines and osteoinductive ones which significantly enhanced osseointegration between the implant and host bone, and next to porous PEEK, probably because of its porous structure [18].

However, there are still some controversies. They suggested that zinc supplementation plays an inhibitory effect on M2 polarization and could switch macrophages toward the M1 phenotype to enhance anticancer activity [102,103]. Therefore, in order to derive a definite therapeutic potential of zinc, more detailed studies need to be carried out. For example, the influence of zinc content in PEEK on macrophage must be taken into consideration.

### 5.3. Calcium-Modified PEEK

Calcium (Ca), an indispensable macro-element of life, is mostly distributed in bones and teeth. It is involved in enzyme reaction, blood coagulation, cardiac muscle contraction, and cell membrane integrity, and influences the osteogenic differentiation of mesenchymal stem cells [104,105,106,107,108]. Furthermore, certain inflammatory signaling pathways are also impacted by calcium, which, in a high concentration, could activate the calcium sensing receptor (CaSR) signaling cascade and then result in the production of Wnt5a, which reduces inflammation by inhibiting the expression of TNF-α through down-regulating NF-kB and TNFR1 via the Wnt5a/ /Ror2 signaling pathway [83,109,110,111,112,113].

Furthermore, calcium is also proved to be involved in macrophage polarization [83,109,114]. Through the mediation of an intermedium (such as norepinephrine), calcium ions were bound on the PEEK surface. Toita R et al. proved that Ca-modified PEEK could induce LPS-stimulated RAW264.7 macrophages towards an anti-inflammatory/wound healing type (M2 phenotype), producing higher levels of anti-inflammatory cytokines but causing lower levels of pro-inflammatory ones when compared to pure PEEK, and subsequently activate osteoblastic cell proliferation and differentiation [10]. This is partly attributed to the calmodulin (CaM) signaling pathway activated by Ca ions, or it is the induction of M2 macrophage polarization by calcium ion modification that is beneficial to enhance the capacities of chemotaxis and the osteoinductivity of BMSCs [83].

Apart from its direct potential in improving newborn formation [115,116], it should be emphasized that the indirect osteogenic function of calcium incorporated in PEEK also results from its osteoimmunomodulation by inducing M2 macrophage polarization. In order to simultaneously obtain and balanc the optimal effects of both, the porous PEEK is probably an excellent carrier which not only avoids the rapid release of calcium by chemical bonding but also the porous structure itself can improve osteogenesis. However, it is necessary to further study how to obtain the maximum osteogenic effect by including the combination of calcium content, porosity, etc.

### 5.4. Phosphate-Modified PEEK

Phosphorus plays an indispensable role in a series of biological processes, such as energy metabolism, bone mineralization, nucleic acid synthesis, phosphorylated enzymes, cell membranes, and cell signaling [117,118]. As reported in the previous studies, β-tricalcium phosphate is an excellent osteoinductive biomaterial which can stimulate macrophages into the M2 phenotype through releasing calcium, cobalt, and magnesium ions, and phosphate may improve the biocompatibility of implants by enhancing osteoblast attachment and differentiation [79,119,120,121]. However, what role phosphate ions play in regulating macrophage polarization has been neglected. 

A recent study carried out by Villa-Bellosta R et al. showed that phosphate ions could induce a higher differentiation of an M2-anti-inflammatory macrophage phenotype which was evidenced by the higher expression of a typical M2 macrophage marker (arginase-1) [122]. In addition, Fukuda N et al. developed a novel PEEK modified with phosphate though plasma treatment and a subsequent phosphorylation reaction [58]. Their results showed that phosphorylated PEEK could lower the levels of TNF-α (a pro-inflammatory cytokine) but raise the levels of IL-10 (an anti-inflammatory cytokine) produced by macrophages when compared with the bare and plasma treated PEEK. Furthermore, they supposed that phosphate ions may play a positive role in inducing M2 polarization. However, the detailed mechanism involved in the phosphate-induced M2 polarization is unclear, which needs to be further explored.

## 6. Challenges and Outlook

The advent of PEEK has had a great influence in the field of medical biomaterial implants, and bio-implants based on PEEK have been considered as a good alternative to titanium-based and ceramic-based implants in spinal, cranial, dental, traumatic, and orthopedic surgeries owing to their excellent radiolucency, stable physicochemical properties, bio-compatibility, and elastic modulus similar to normal human bones [36,37,123,124,125]. However, it is the fact that PEEK is biologically inert that, to some extent, limits the clinical application of PEEK due to preventing an excellent bonding with surrounding bone cells and tissues when implanted into host bone [126]. Presently, direct surface modification, deposition techniques, and bioactive composite fabrication play important roles in improving the bioactivity of PEEK, which provides PEEK with the required osteoimmunomodulation involved in regulating immune cells including macrophages, T cells, B cells, mast cells, etc. [18,19,22,127,128]. Disappointingly, as far as the authors know, the majority of PEEK-based biomaterials have not been approved and put into clinical use. With regard to this, there exist three major barriers to the translation of PEEK-based biomaterials from the bench to the bedside. Firstly, it is a question of whether the excellent mechanical properties of PEEK are compromised by the presence of porous structure and additional bioactive substances after being processed by the aforementioned techniques [49,129,130]. Secondly, how to controllably release bioactive composites from PEEK substrate for obtaining a durable and desirable effect deserves to be studied. Thirdly, it is discouraging that the clinical approval and the preliminary researches of novel PEEK-based biomaterial implants are very time-consuming and costly [131].

Recently, in order to address these issues, many efforts have been made. For example, the incorporation of carbon fiber provides PEEK material with a good mechanical strength comparable to that of human cancellous bone, which is relatively strong and safe to be implanted in vivo [132,133], which may be a better choice than the binding between PEEK and bioactive composites through some intermedi without changing the inherent structure of PEEK [10]. On the other hand, the layer-by-layer assembled strategy can allow PEEK-based biomaterials a controlled and durable release of bioactive composites [134,135]. Therefore, through advanced technologies, PEEK not only keeps the acceptable mechanical properties but is also endowed with the needed bioactive potential, and it is promising that PEEK, with the development of advanced technology, could pave the way for the advent of medical biomaterial implants.

Apart from the aforementioned studies focusing on the osteogenic effect of bioactive substances in combination with PEEK by regulating macrophage polarization, the phenotypes of macrophages are also modulated by biomaterial topography, bioactive elements, cytokines, RNAs, and chemical substances, such as nanostructure surface, strontium (Sr), IL-10, IL-4, lncRNA-MM2P, chitosan, curcumin, etc. [34,136,137,138,139,140,141]. If these are used as the modification of PEEK, PEEK may have worldwide application in the medical field because of its great potential for modulating M2 polarization to improve osteogenesis. However, this issue related to bioactive substance incorporation is highly complicated and still needs to be addressed.

In the next few years, two strategies need to be implemented. On one hand, seeking a novel technique provides PEEK with a perfect design that guarantees its own good properties and endows it with more excellent osteoimmunomodulation. On the other hand, the complex mechanisms of macrophage polarization of bio-activated PEEK need to be further understood, which contributes to guiding the design of bio-activated PEEK in improving osteoimmunomodulation.

## 7. Conclusions

PEEK can cause a macrophage response, and it is clear that different macrophage phenotypes play different roles in the tissue repairing process. The timely modulation of macrophage phenotypes seems to be an important aspect of improving osteogenesis through the fabrication of PEEK topography and the incorporation of bioactive substances. Presently, many bioactive substances have been studied and have shown their osteoimmunomodulation by modulating macrophage polarization. In addition, of these bioactive substances, several have been used to modify PEEK, and excellently bio-activated PEEK can enhance M2 macrophage phenotypes to promote new bone formation surrounding implants. In the future, bio-activated PEEK with modulation of the type of immune environment to induce osteogenesis should be focused on, and the specific mechanisms should also be better understood. This will shed light on the development of an optimally bio-activated PEEK with more favorable osteoimmunomodulation.

## Figures and Tables

**Table 1:** Technologies of developing porous PEEK.

Technologies	References
Using sulfonation and subsequent water immersion	[18,44,45,46]
Using a weaving technology	[47]
Using a melt extrusion and porogen leaching process	[48,49,50,51,52]
Using PEEK powder and a particulate leaching technique	[53,54]
Using a computer-aided design program and then printed via selective laser sintering (SLS)	[55,56]

**Table 2:** The mechanical properties of PEEK and SPEEK.

Sample	Breaking Point (N)	Elasticity Modulus (N/mm^2^)	Fracture Strength (N/mm^2^)
PEEK	3425.20	4487.36	94.3143
SPEEK	3476.50	4681.34	95.7271

## Data Availability

Not applicable.
